# Identification of a novel sepsis prognosis model and analysis of possible drug application prospects: Based on scRNA-seq and RNA-seq data

**DOI:** 10.3389/fimmu.2022.888891

**Published:** 2022-10-28

**Authors:** Haihong He, Tingting Huang, Shixing Guo, Fan Yu, Hongwei Shen, Haibin Shao, Keyan Chen, Lijun Zhang, Yunfeng Wu, Xi Tang, Xinhua Yuan, Jiao Liu, Yiwen Zhou

**Affiliations:** ^1^ Department of Emergency Laboratory, Clinical Laboratory Medical Center, Shenzhen Hospital, Southern Medical University, Shenzhen, China; ^2^ Department of General Surgery, Shenzhen Hospital, Southern Medical University, Shenzhen, China

**Keywords:** sepsis, scRNA-seq, risk score, immune cell, prognosis model

## Abstract

Sepsis is a disease with a high morbidity and mortality rate. At present, there is a lack of ideal biomarker prognostic models for sepsis and promising studies using prognostic models to predict and guide the clinical use of medications. In this study, 71 differentially expressed genes (DEGs) were obtained by analyzing single-cell RNA sequencing (scRNA-seq) and transcriptome RNA-seq data, and Gene Ontology (GO) and Kyoto Encyclopedia of Genes and Genomes (KEGG) enrichment pathway analyses were performed on these genes. Then, a prognosis model with CCL5, HBD, IFR2BP2, LTB, and WFDC1 as prognostic signatures was successfully constructed after univariate LASSO regression analysis and multivariate Cox regression analysis. Kaplan–Meier (K-M) survival analysis, receiver operating characteristic (ROC) time curve analysis, internal validation, and principal component analysis (PCA) further validated the model for its high stability and predictive power. Furthermore, based on a risk prediction model, gene set enrichment analysis (GSEA) showed that multiple cellular functions and immune function signaling pathways were significantly different between the high- and low-risk groups. In-depth analysis of the distribution of immune cells in healthy individuals and sepsis patients using scRNA-seq data revealed immunosuppression in sepsis patients and differences in the abundance of immune cells between the high- and low-risk groups. Finally, the genetic targets of immunosuppression-related drugs were used to accurately predict the potential use of clinical agents in high-risk patients with sepsis.

## Introduction

Sepsis causes life-threatening organ dysfunction due to dysregulated host response to infection (1, 2). The tissue and organ damage caused by sepsis can be attributed to excessive activation of the inflammatory response, immune dysfunction, and coagulation disorders (3). There are significant differences in the primary sources of sepsis, with community infections accounting for 60%–70% of all cases, 20%–30% of cases in non-intensive care units (non-ICUs), and 5%–9% of primary cases acquired in the ICU (4, 5). Mortality rates vary considerably from region to region, with a combination of social, economic, political, health, and even climatic factors influencing the epidemiological data on sepsis, which still approaches 60% in some developing countries (6), while in developed countries, the mortality rate is usually reported as 20%–30% (7–9).

The ability of biomarkers to diagnose sepsis and determine its severity often falls short of expectations. Many of the classical biomarkers associated with the degree of the inflammatory response, such as interleukin-6 (IL-6), interleukin-10 (IL-10), C-reactive protein (CRP), platelet (PLT), and procalcitonin (PCT), have been shown to correlate well with sepsis severity and clinical outcome in population-based studies (10, 11). However, the clinical application of these markers is largely reflected in a commonality of an inflammatory response to early disease and a lack of specificity, so their use in the progression of sepsis is greatly compromised. With advances in molecular biology, biomarkers also include chemokines, damage-associated molecular patterns (DAMPs), endothelial cell markers, leukocyte surface markers, non-coding RNAs, miRNAs, soluble receptors, and alterations in metabolite and gene expression (transcriptomics). A series of new biomarkers, such as heat shock proteins (HSPs), high mobility group box 1 (HMGB-1), C-C motif chemokine ligand 2 (CCL2), C-X-C motif chemokine ligand 10 (CXCL10), S100 calcium binding protein B (S100B), intercellular adhesion molecule 1 (ICAM-1), and E-selectin (12, 13), are closely associated with the progression of sepsis. Biomarkers can classify patients with sepsis into biological phenotypes, such as hyperinflammatory versus immunosuppressive (14). However, studies have demonstrated a lack of strong evidence for the ability of these markers to predict the prognosis of sepsis (7).

The incidence of sepsis in China is about 200–270 per 100,000 people (8), and has become one of the most economically burdensome diseases for society and families, with a poor prognosis and a persistently high mortality rate (15). To date, there is a lack of prognostic models with ideal biomarkers for clinical application. As single-cell sequencing is increasingly used in various diseases, multi-omics studies will be more beneficial for disease diagnosis and prognosis, and models will be constructed with greater precision and reliability. In the current study, we focused on obtaining differentially expressed genes (DEGs) between septicemic and healthy individuals using RNA-seq and scRNA-seq data from the GEO database, and carried out GO and KEGG enrichment analyses. The co-expressed DEGs were subjected to univariate LASSO regression analysis, multivariate Cox regression analysis, construction of a predictive signature for sepsis, KM survival analysis, ROC analysis, nomogram survival charts, internal validation, and principal component analysis (PCA) to further validate the diagnostic and prognostic value of the model in sepsis. We analyzed the differences in immune cell abundance between high- and low-risk groups and gene set enrichment analysis (GSEA) to explore possible mechanisms. Finally, we combined scRNA-seq data with gene targeting of immunosuppressive-related drugs to accurately predict the future of immune-targeted drugs in sepsis and its high-risk patients.

## Materials and methods

### Patients and datasets

Both scRNA-seq data and transcriptomic RNA data for sepsis were obtained from the GEO database. GSE167363 was the scRNA-seq data, with 12 subsets included in the study, containing data from 2 healthy individuals and 10 patients with sepsis (5 survived and 5 died). GSE65682 is RNA-seq data, and a total of 802 people were included in the study, of whom 42 were healthy, 760 had sepsis, and 479 had survival status.

### Analysis of DEGs

The 12 subsets of GSE167363 scRNA-seq data were integrated and analyzed using R software, and single-cell profiles of normal and sepsis groups were constructed, followed by identification of DEGs. DEGs from the GSE65682 RAN dataset were identified by the GEO2R (http://www.ncbi.nlm.nih.gov/geo/geo2r/) tool that comes with the GEO database. The cutoff value for DEGs of both datasets were | log2 fold change (log2 FC) | > 1 and *p*-values < 0.01, and DEGs common to both datasets were defined as hub-DEGs and visualized by Venn diagrams.

### Functional analysis

Gene function analysis is often considered an important part of the translation of molecular research results from high-throughput methods to biological significance. Statistical analysis of gene function was performed using the clusterProfile package, and bubble plots were used to visualize the functional profiles of DEGs containing GO and KEGG. *p* < 0.05 was considered statistically significant.

### Construction of the prognostic signatures

Univariate Cox regression analysis was performed on each of the co-expressed DEGs to screen for genes significantly associated with sepsis overall survival (OS) in the GSE65682 dataset. These identified genes were then subjected to LASSO Cox regression analysis and multivariate Cox regression analysis to construct a multivariate model of genes associated with sepsis prognosis. The formula used to calculate the prognostic risk score for the analysis of each patient is as follows: risk score = gene1 expression level × i1 + gene2 expression level × i2 +…+ geneN expression level × iN, where i represents the coefficient value. Using the median value of the risk score as a cutoff, patients with sepsis were divided into high- and low-risk groups. We randomly distinguished patients into a train set and a test set by setting a head count ratio value of 1:1, which was used for internal validation.

### Patient enrollment and quantitative real-time PCR

Patients diagnosed with sepsis in the ICU department of Shenzhen Hospital of Southern Medical University from 31 July 2022 to 5 September 2022 were enrolled. Enrollment criteria were as follows (1): age: 18–90 years old, and (2) patients met sepsis 3.0 diagnostic criteria. We excluded patients who have been discharged or who have died within 24 h after admission; those who needed emergency surgery after admission; those who participated in other clinical research; those with tumors, autoimmune diseases, and immunodeficiencies; or those on long-term immunosuppressant therapy. 

RNA was extracted from whole blood using TRIzol reagent (12183-555, Invitrogen) following the manufacturer’s instructions. Prime-Script RTase (Takara) was used for reverse transcription. With the help of the premix Ex-Taq (Takara), the gene expression level was determined by qPCR and normalized to the glyceraldehyde-3-phosphate dehydrogenase (GAPDH). We used the 2^−ΔCT^ method to calculate the expression level. The primer pairs used in the experiments are listed in **Data S1**. The study was approved by the Ethics Committee of Shenzhen Hospital, Southern Medical University (Registration number: MCSC-20220909-0001).

### Construction and validation of the nomogram

We combined risk scores with clinical characteristics of age, sex, ICU-acquired infection, pneumonia, and diabetes to construct nomogram survival charts that predicted 7-, 14-, and 21-day OS in patients with sepsis. Calibration curves were used to test the agreement of predicted survival with actual survival.

### Principal component analysis and GSEA

Patients were divided into high-risk and low-risk groups based on risk scores. Differences in gene expression profiles between patients in the low- and high-risk groups were verified by PCA. Identification of relevant pathways and molecular mechanisms in high- and low-risk groups in a cohort of patients with sepsis by GSEA (https://www.gsea-msigdb.org/gsea/index.jsp). We visualized the top five GO and KEGG pathways for positive and negative correlations. *p* < 0.05 and false discovery rate (FDR) < 0.25 were considered to be statistically significant thresholds.

### Immune infiltration analysis

The CIBERSORTx tool is used to estimate gene expression profiles and to estimate the abundance of member cell types in mixed cell populations using gene expression data (16). We used the gene expression matrix for 22 classes of immune cells provided on the website in combination with our sample gene expression matrix data to compare immune infiltration and function in the high- and low-risk groups using a two-sample Wilcoxon test. The expression abundance of each cell type in the normal, sepsis survivor, and sepsis death groups was analyzed by scRNA-seq data.

### Statistical analysis and R package

All statistical analyses were performed using R software (version 4.0.2). The Seurat and harmony packages were used mainly for integration analysis of single-cell sequencing data and removal of batch effects. The limma package was used mainly for the identification of DEGs. The Survival, survminer, glmnet, caret, timeROC, and survivalROC packages were used for univariate LASSO regression analysis, multivariate Cox regression analysis, construction of survival curves, ROC curves, and determination of area under the curve (AUC) values. The rms package is used for the construction of column line tables and calibration plots. *p* < 0.05 and *p* < 0.01 are considered to be statistically significant differences.

## Results

### Single-cell sequencing analysis of peripheral blood cell composition in patients with sepsis

**Figure 1:**
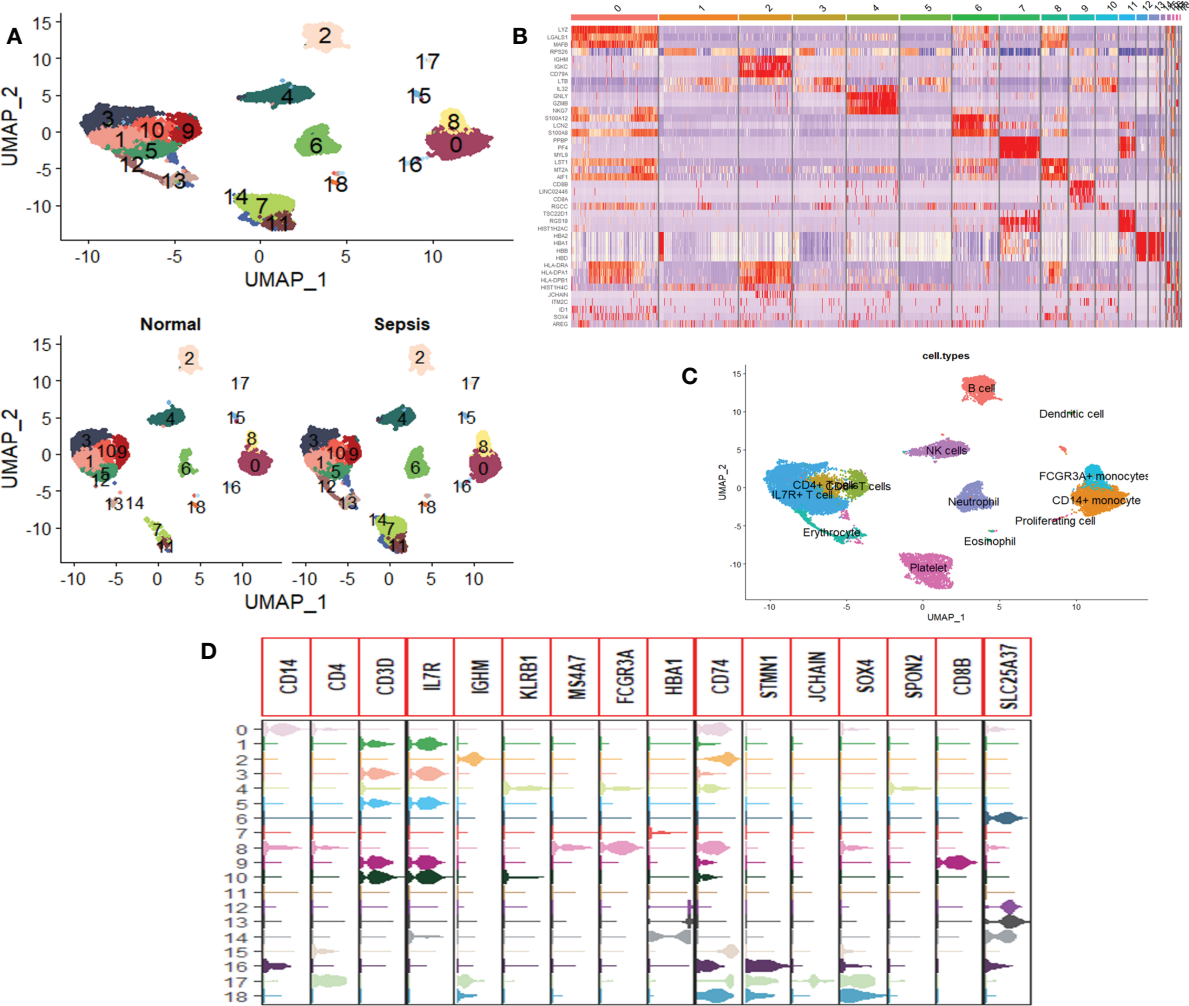
Single-cell RNA sequencing profiles of peripheral blood cells from patients with sepsis. **(A)** UMAP map consisting of 18,462 cells in 19 clusters, namely, 5,257 cells in healthy individuals and 13,205 cells in sepsis patients. **(B)** Heat map visualization of the top three marker genes for each cluster of cells. **(C)** Annotation of peripheral blood cells showing a total of 13 blood cell compositions. **(D)** Violin plots show the distribution of the 16 typical immune cell marker genes in each cell cluster, again validating the accuracy of the annotation.

Twelve subsets of single-cell data were integrated into a dataset containing 20,696 genes, and 18,462 cells, clustered into 19 cell groups by the Uniform Manifold Approximation and Projection (UMAP) method of the Seurat package. There were 5,257 cells in healthy people and 13,205 cells in sepsis patients (**Figure 1A**). Identification and visualization of marker genes in clustered cells were carried out using the FindAllMarkers function (**Figure 1B**). A total of 13 cell types were identified by marker gene combinations combined with a review of the HPC database combined with typical cell marker genes (**Figures 1C, D**).

### Identification of hub-DEGs and functional analysis

**Figure 2:**
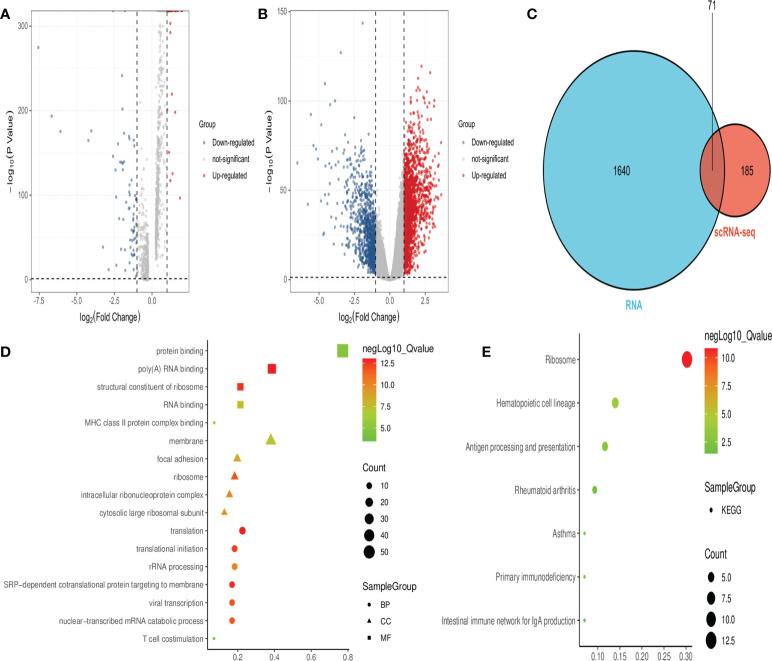
Obtaining hub-DEGs and their GO and KEGG enrichment analysis by analyzing scRNA-seq and RNA-seq datasets. **(A)** Volcano plot showing DEGs between healthy and sepsis populations in the scRNA-seq dataset. Red dots represent upregulated genes and blue dots represent downregulated genes. **(B)** Volcano plot showing DEGs between healthy and sepsis populations in the RNA-seq dataset. Red dots represent upregulated genes and blue dots represent downregulated genes. **(C)** Venn diagram showing 71 hub-DEGs co-expressed in both datasets. **(D)** Bubble plots showing the results of top five GO functional enrichment of 71 hub-DEGs. BP is shown in circles, CC is shown in triangles, and MF is shown in squares, with the horizontal coordinates representing ratio. **(E)** Bubble plots showing the results of the KEGG signaling pathway of 71 hub-DEGs, and the horizontal coordinate represents ratio.

Analysis of the scRNA-seq dataset identified 256 DEGs between healthy individuals and septicemic patients (**Figure 2A**), while a total of 1,711 DEGs were identified in the RNA-seq dataset (**Figure 2B**), with a total of 71 genes co-expressed in both datasets, which are considered hub-DEGs (**Figure 2C**). We continue to analyze GO functional enrichment and KEGG analysis of hub-DEGs through the DAVID web tool. In the BP category, the enrichment was mainly in translation and translational initiation; in the CC category, the enrichment was mainly in membrane and focal adhesion; and in the MF category, the enrichment was mainly in protein binding poly(A) RNA binding (**Figure 2D**). In the signaling pathway analysis, the KEGG pathways identified for these candidate genes were ribosome, hematopoietic cell lineage, antigen processing, and presentation (**Figure 2E**).

### Construction of the hub-DEG prognostic signatures and experimental validation of expression in sepsis patients

**Figure 3:**
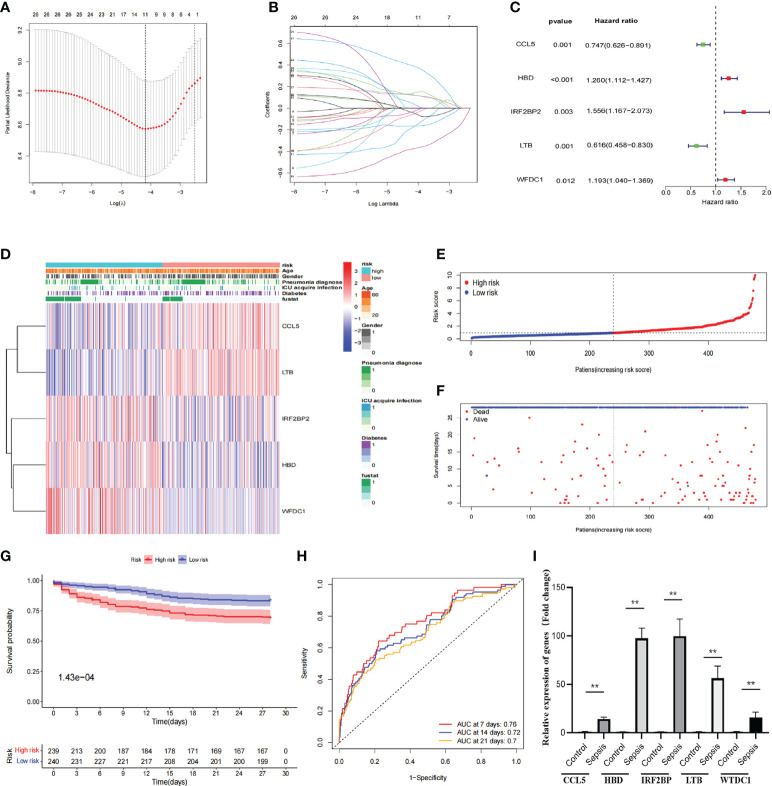
Construction of prognostic signatures by hub-DEGs. **(A)** Lasso regression analysis showing that 11 genes are strongly associated with OS in sepsis. **(B)** Curve diagram showing the calculated regression coefficients for the hub-genes. **(C)** Forest plot showing five genes (CCL5, HBD, IRF2BP2, LTB, and WFDC1) as prognostic signatures of the prognosis model after multivariate Cox regression analysis. **(D)** Heat map showing the expression of five prognostic risk genes between the high- and low-risk groups. CCL5 and LTB were higher in the low-risk group than in the high-risk group, while IRF2BP2, HBD, and WFDC1 were lower in the low-risk group than in the high-risk group. **(E, F)** Distribution of risk scores for sepsis patients and correlation between risk scores and survival data in scatter plots. Patients in the high-risk and dead groups are indicated by red dots and those in the low-risk and alive groups are indicated by green dots. **(G)** K-M survival curves, analysis of OS time curves between the high-risk and low-risk groups. Patients in the high-risk group had a significantly higher 28-day survival rate than those in the low-risk group. **(H)** ROC curves showing AUC values for predicting signed 7-, 14-, and 21-day survival. The AUC curves for 7, 14, and 28 days are shown as red, green, and orange lines, respectively. **(I)** The expression of five genes (CCL5, HBD, IRF2BP, LTB, and WTDC1) in peripheral blood was compared between healthy controls and sepsis patients by quantitative real-time PCR. Differences between two groups were analyzed using the *t*-test (**p < 0.001).

Hub-DEGs identified 11 significant genes that were strongly associated with OS after univariate and LASSO Cox regression analyses (**Figure 3A**), and the regression coefficients for these significant genes are calculated in **Figure 3B**. Multivariate Cox regression analyses showed that five genes (CCL5, HBD, IRF2BP2, LTB, and WFDC1) were identified as construct prognostic signatures (**Figure 3C**). The risk score is calculated using the following formula: risk score = (−0.292 × CCL5 expression) + (0.231 × HBD expression) + (0.442 × IRF2BP2 expression) + (−0.484 × LTB expression) + (0.177 × WFDC1 expression). The risk score was calculated for each patient according to the formula and the median risk score was used as the threshold to classify patients into high- and low-risk groups. The heat map shows the expression of the five prognostic risk genes between the high-risk and low-risk groups (**Figure 3D**). The distribution of risk scores for patients with sepsis and the correlation between risk scores and survival data are shown in the scatter plot (**Figures 3E, F**). Subsequently, K-M survival curves were plotted and the OS time profiles of the high-risk and low-risk groups were analyzed to determine the ability of the model to predict the clinical prognosis of patients with sepsis, and the results showed that patients in the high-risk group had significantly lower 28-day OS than those in the low-risk group, *p* = 1.43e-4 (**Figure 3G**). The AUC values for 7-, 14-, and 21-day OS were 0.76, 0.72, and 0.7, respectively, indicating good predictive performance of the risk score (**Figure 3H**). The expression of five genes was compared between healthy controls (*n* = 5) and patients with sepsis (*n* = 20) using quantitative real-time PCR. The results showed that the expression of all these genes was significantly higher than that in the healthy control group (**Figure 3I**).

### Risk score as an independent risk factor for prognosis of sepsis

**Figure 4:**
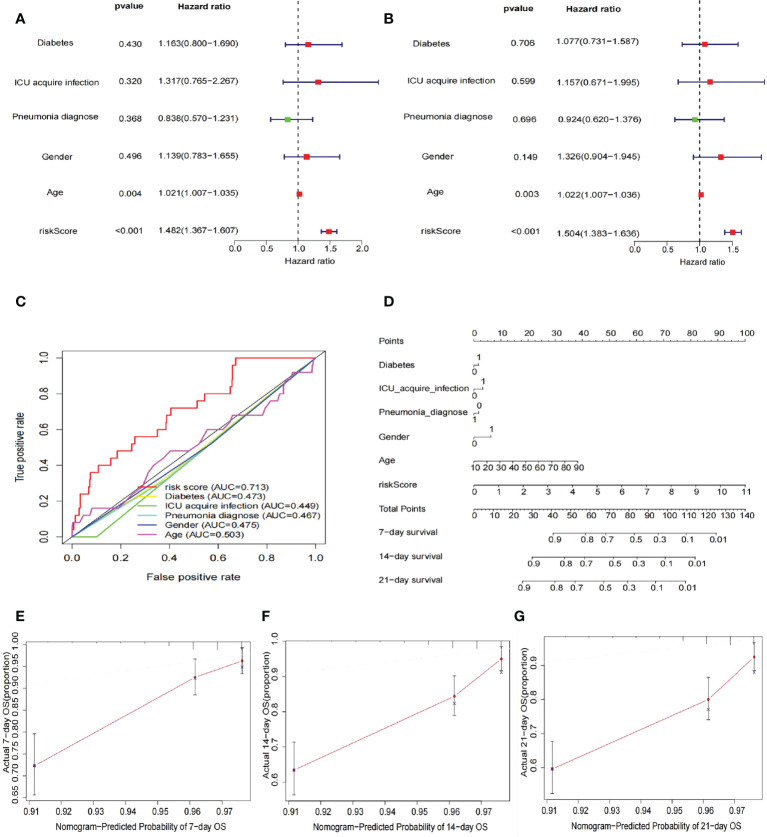
Risk score as an independent risk factor for prognosis of sepsis. **(A)** Forest plot showing results of univariate Cox regression analysis after inclusion of clinical clinicopathological characteristics (age, sex, pneumonia, ICU-acquired infection, and diabetes). The results show that the risk score has a good predictive value. **(B)** The forest plot shows the results of the multivariate Cox regression analysis, with statistically significant *p*-values for the two indicators risk score and age. **(C)** ROC curves showing the AUC values of clinicopathological characteristics and risk scores. The AUC value for the risk score was 0.713, much greater than the other clinicopathological characteristics. **(D)** Construction of nomogram plots of clinicopathological characteristics and risk scores. The risk score showed good predictive value at 7, 14, and 21 days for sepsis. **(E–G)** Calibration curves showing the agreement between actual OS rates and predicted 7-, 14-, and 21-day survival rates.

After inclusion of clinicopathological characteristics (age, gender, pneumonia, ICU-acquired infection, and diabetes), both univariate Cox regression analysis (HR = 1.482, 95% CI = 1.367–1.607, *p* < 0.001) and multivariate Cox regression analysis (HR = 1.504, 95% CI = 1.383–1.636, *p* < 0.001) found model risk score as an independent prognostic indicator for patients with sepsis (**Figures 4A, B**). We visualized the differences in clinicopathological variables between the high- and low-risk groups by heat map and only found differences in age (*p* < 0.05) as well as risk score (*p* < 0.05) between the high- and low-risk groups (**Figure 4C**). The AUC value of risk score was 0.713, significantly higher than age (0.503), gender (0.475), pneumonia (0.467), ICU-acquired infection (0.449), and diabetes (0.473), indicating that the risk score was superior to the clinicopathological variables in predicting the prognosis of patients with sepsis (**Figure 3D**). To further predict the prognosis of sepsis, we constructed a nomogram containing clinicopathological variables and risk scores that predicted the prognosis of patients with sepsis at 7, 14, and 21 days (**Figure 4D**). The calibration curves showed a high degree of agreement between actual OS rates and predicted 7-, 14-, and 21-day survival rates (**Figures 4E–G**).

### Relationship between prognostic signatures and prognosis of patients with sepsis under different clinicopathological variables

**Figure 5:**
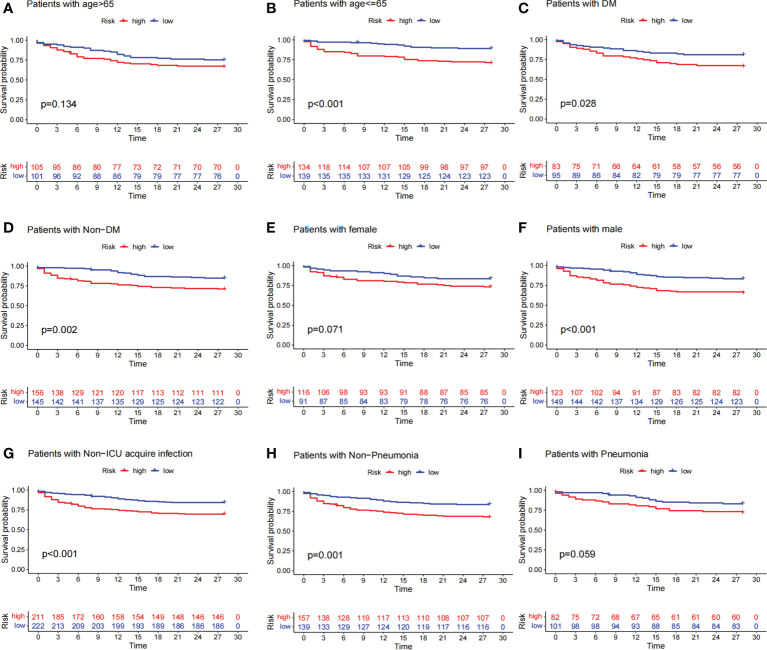
Survival curves for clinicopathological variables. **(A–I)** Kaplan–Meier survival curve shows the relationship between high- and low-risk groups and sepsis prognosis under different clinicopathological variables. The high-risk group is shown in red and the low-risk group is shown in blue. The data showed that patients in the high-risk group had lower survival rates than those in the low-risk group across different clinicopathological variables.

To investigate the relationship between predictive signature and prognosis in patients with sepsis classified by different clinicopathological variables, patients with sepsis were grouped by age, diabetes, gender, ICU-acquired infection, and pneumonia. Among the clinicopathological groups, patients in the high-risk group had significantly shorter OS than those in the low-risk group (**Figure 5**). These results suggest that clinicopathological variables have little effect and that prognostic signatures can predict the prognosis of patients with sepsis.

### Internal verification of the prognostic signatures

**Figure 6:**
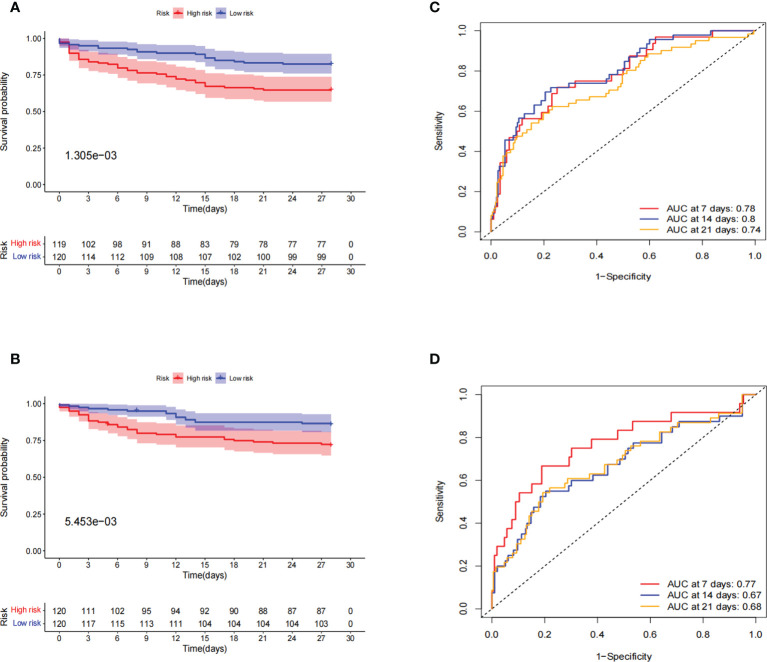
Internal validation of prognostic signatures for the entire sepsis dataset. **(A, B)** Kaplan–Meier survival curve in the train and test cohorts. Patients in the high-risk group had a significantly lower number of days of survival than those in the low-risk group. The red and blue lines represent the high-risk and low-risk groups, respectively. **(C, D)** ROC curve and AUCs at 7-, 14-, and 21-day survival in the train and test cohorts. Both the train and test cohorts showed better predictive performance, especially at 7 days, with the highest AUC values of 0.78 and 0.77, respectively.

To verify the applicability of the dataset, we performed an internal validation of the data. The entire dataset samples were randomly assigned in a 1:1 ratio to the train cohort (240 cases) and test cohort (239 cases). In the train set and test set, the 28-day OS rate was significantly lower in the high-risk group than in the low-risk group, and their *p*-values were 1.305e-3 and 5.453e-3, respectively (**Figures 6A, B**). Further ROC curve analysis showed better predictive performance in both the train and test groups, especially in the early stages of the disease. In the train cohort, the AUCs for 7-, 14-, and 21-day OS were 0.78, 0.8, and 0.74, respectively (**Figure 6C**). In the test cohort, the AUCs for 7-day, 14-day, and 21-day survival were 0.77, 0.67, and 0.68, respectively (**Figure 6D**).

### PCA and GSEA

**Figure 7:**
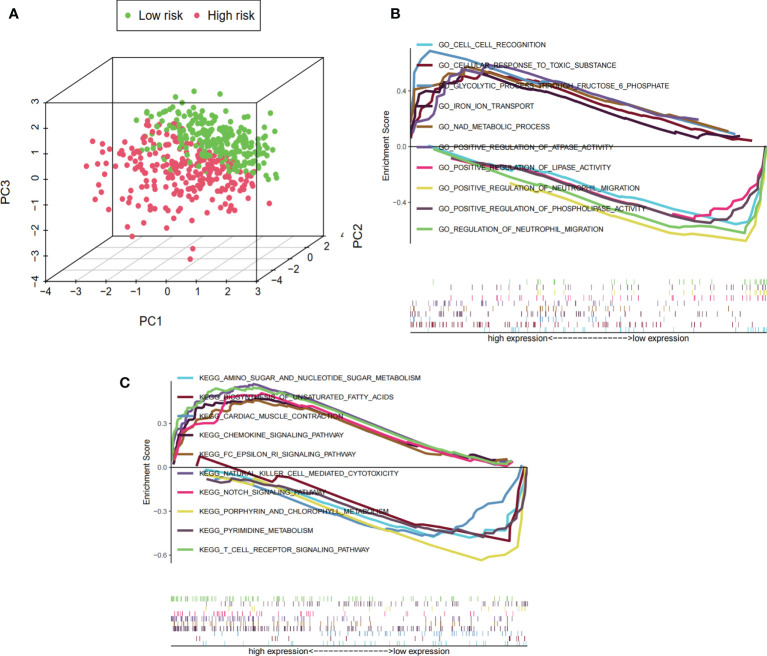
PCA and GSEA based on risk score groupings. **(A)** PCA distribution of clinical samples based on high- and low-risk groups. The three-dimensional graphs distinguish well between the distribution of patients in the high- and low-risk groups, where the distribution of the sample in the high-risk group is represented by red dots and the distribution in the low-risk group is represented by green dots. **(B)** GSEA showing the results of GO functional enrichment analysis for the high- and low-risk groups. The value 0 of the enrichment score is used as the cutoff value to show the top five GO functional enrichment analysis for high and low expression, respectively. **(C)** GSEA showing the results of KEGG signaling pathway enrichment analysis in the high- and low-risk groups. The value 0 of the enrichment score is used as the cutoff value to show the top five KEGG signaling pathway analysis for high and low expression, respectively.

The differences in the prognostic model constructed by predictive signature between the low-risk and high-risk groups were detected by PCA. Based on the results of the prognostic model, the low-risk and high-risk groups were clearly distinguished, which verified that the predictive signature could better distinguish the low- and high-risk groups (**Figure 7A**). Because of the significant differences in prognosis between patients in the high-risk and low-risk groups, we conducted the GSEA study to examine the differences in GO and KEGG between the high-risk and low-risk groups. We found that in the GO functional analysis, cell recognition, cellular response to toxic substance, glycolytic process through fructose-6-phosphate, iron ion transport, and NAD metabolic processes were significantly enriched in the high-risk group (**Figure 7B**). In the KEGG analysis, amino sugar and nucleotide sugar metabolism, biosynthesis of unsaturated fatty acids, cardiac muscle contraction, chemokine signaling pathway, and Fc epsilon RI signaling pathway were significantly enriched in the high-risk group (**Figure 7C**).

### Immune cell and drug target gene analysis

**Figure 8:**
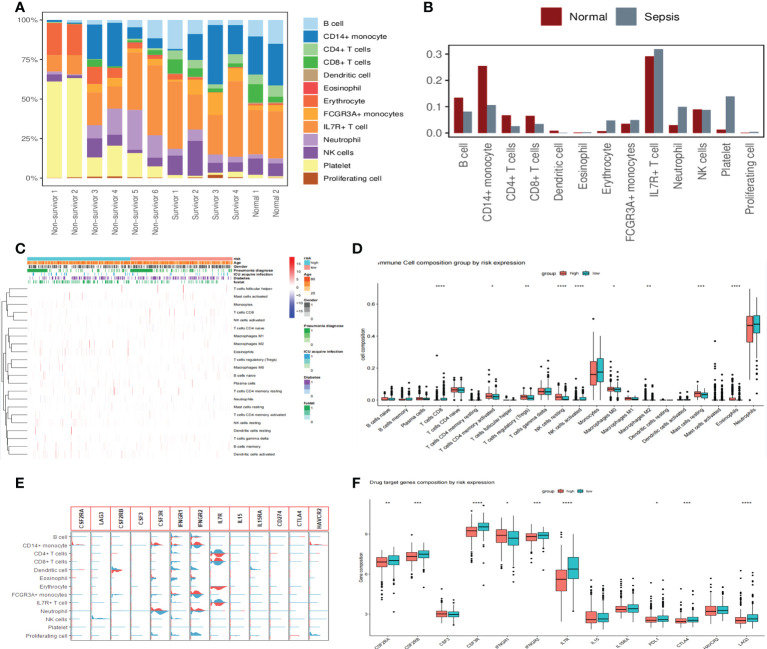
Changes in immune cells and drug target gene analysis in patients with sepsis. **(A)** Proportions of 13 immune cell species in 12 scRNA-seq datasets. The proportion of immune cells is significantly reduced in non-survivor sepsis patients. **(B)** The proportional distribution of 13 immune cell types in normal and sepsis populations and the differences between them. The B cells, CD14+ monocytes, CD4+ T cells, CD8+ T cells, dendritic cells, and NK cells were all higher in the normal group than in the sepsis group. **(C)** Heat map showing the correlation analysis of different clinicopathological features with 22 immune cell landscapes, where we used the high- and low-risk groups as the main differentiating boundary. **(D)** Further study of 22 immune cells between the high- and low-risk groups, with an asterisk (*) sign indicating a significant difference and more asterisks indicating more significant cell differences. **(E)** Violin diagram showing the expression of 13 drug target genes in 13 cell clusters. Light blue denotes the normal group and red indicates the sepsis group. **(F)** The distribution of immune function-related drug target genes in high- and low-risk populations showed that nine target genes differed between high- and low-risk groups.

We showed that immune cells differed between healthy and sepsis patients by analyzing scRNA-seq data from 12 patients, where B cells, CD14+ monocytes, CD4+ T cells, CD8+ T cells, dendritic cells, and NK cells were higher in the normal group than in the sepsis group, indicating the presence of immunosuppression in the latter (**Figures 8A, B**). To further explore the relationship between sepsis prognosis and immune cells, we quantified 22 immune cell scores in the high-/low-risk group using the CIBERSORTx algorithm. The relationship between different clinicopathological characteristics and immune landscape is shown by the heat map in **Figure 8C**. We found that a total of nine immune cells differed between the two groups, with CD4 memory activated T cells, regulatory T cells (Tregs), resting NK cells, M0 macrophages, M2 macrophages, resting mast cells, and eosinophils higher in number, while in the low-risk group, CD8 T cells and activated NK cells were higher in number (**Figure 8D**). These results suggest that patients in the high-risk group showed a significant decrease in immune cytotoxicity and a significant increase in the regulation of humoral immunity. Analysis of drug targets of immune function-related drugs predicted the future of immune-targeted drugs in sepsis, and the results showed that nine target loci (CSF2RA/B, CSF3R, IFNGR1/2, IL7R, PDL1, CTLA4, and LAG3) were higher in sepsis than in healthy populations, as well as differences in high-/low-risk groups (**Figures 8E, F**).

## Discussion

Sepsis is a systemic inflammatory response syndrome (SIRS) caused by bacterial, fungal, and viral infections, which can lead to multi-organ dysfunction and is a highly lethal clinical disease (17, 18). The ideal biomarker should possess features such as affordability, utility, and the ability to achieve a highly specific and sensitive diagnosis of sepsis. Although hundreds of markers are clinically available for the evaluation of sepsis, many are not used in clinical practice because of their lack of sensitivity and specificity (19–21). No single biomarker can diagnose, predict, and follow sepsis treatment, and the real challenge is to select the best and validate clinically useful biomarker models from a large number of biomarkers (22). In recent years, studies have been conducted to construct the prognosis of sepsis from RNA-seq data (4, 23, 24). However, the study of constructing sepsis prognosis by scRNA-seq combined with RNA-seq data has not been reported, and the present study fills this gap.

Using scRNA-seq and RNA-seq data, we identified 71 hub-DEGs that were significantly different in sepsis and normal subjects. After GO and KEGG analysis, these genes were mainly associated with ribosome, hematopoietic cell lineage, and immune dysfunction. Immediately after, for these hub-DEGs, using univariate and LASSO Cox regression analysis, 11 significant genes were associated with OS, and finally, multivariate Cox regression analysis showed that 5 genes (CCL5, HBD, IRF2BP2, LTB, and WFDC1) were identified as the prognostic signatures. CCL5 not only acts as a chemokine involved in the inflammatory response and mediates the wandering and infiltration of immune cells, but also regulates cell growth and differentiation (25). Studies have shown that the expression of CCL5 is significantly higher in septic patients than in non-sepsis patients and is also strongly associated with the prognosis of sepsis (26, 27). HBD encodes the hemoglobin δ chain, and HbA2 is a tetramer composed of two α and δ bead chains, which account for 2%–3% of the total in normal individuals (28). Anemia symptoms are also one of the important clinical manifestations of sepsis. It has also been shown that some viral infections can alter the ratio of HbA2, and even the recently prevalent COVID-19 infection can cause changes in HbA2, but the mechanism is not clear (29–32). Animal models of sepsis have shown that cardiac overexpression of IRF2BP2 effectively inhibits sepsis-induced cardiac dysfunction, inflammatory response, and cell death through activation of the AMPK signaling pathway (33). During sepsis, lipopolysaccharide (LPS) activates the pro-inflammatory effects of the miR-155-5p/IRF2BP2/NFAT1 axis, leading to loss of lung or heart function (34). Knockdown of IRF2BP2 cells promoted the binding of IRF2 to the PD-L1 promoter, which, in turn, inhibited PD-L1 promoter activity and suppressed PD-L1 expression (35). LTB anchors lymphotoxin to the cell surface through the formation of heterogeneous trimers. The predominant form on the lymphocyte surface is one LT-α binding two LT-β to form a complex that is the primary ligand for the LT-β receptor (36). LTB is an inducer of the inflammatory response system and is involved in the normal development of lymphoid tissue, which, in turn, affects the immune function of the body (37). Studies have shown that LT-α was tested in sepsis patients’ sera, and compared to normal volunteers, LT-α was detected in 33% of sepsis sera, and 16% of normal sera. There was no difference in LT-α in sepsis sera even when grouped by pathogen type (38). Recent findings show that SNP LTA +252 is associated with the development of sepsis (39). Although studies have shown a direct correlation between LT-α and sepsis, direct evidence for a correlation between LTB and sepsis is lacking in studies. WFDC1 is a whey acidic protein four-disulfide core member that exhibits diverse growth and immune-associated functions *in vitro*. WFDC1 is a key regulator of the inflammatory response and may be associated with macrophage recruitment (40). In addition, it is involved in the regulation of memory T cells during human immunodeficiency virus infection (41). Except for HBD, all of the above four prognostic signature genes are closely related to the regulation of immune function in the organism. Our study showed that all five prognostic signature genes were significantly higher in sepsis patients than in normal subjects.

The ROC curves showed that the prognostic signatures had good predictive performed and performed best in the early stages of the disease. The nomogram survival charts show that the prognosis of sepsis is strongly correlated with two elements, risk score and age, and poorly correlated with gender, diabetes, ICU-acquired infection, and pneumonia. Prognostic signatures appear more reliable and precise than clinicopathological variables in predicting the prognosis of patients. We also found that the prognostic signatures predicted the prognosis of sepsis, conditional on the exclusion of relevant clinicopathological variables. The prognostic signatures also have good predictive performance as verified by internal validation. PCA validation shows that the prognostic signatures can distinguish well between high- and low-risk group sample distributions.

Subsequently, we performed GO and KEGG enrichment analysis by the GSEA method, and GO was mainly enriched in cell–cell recognition, cellular response to toxic substance, glycolytic process through fructose 6-phosphate, iron ion transport, and NAD metabolic process. All five of these GO functions are closely related to sepsis (42–45). The results of KEGG analysis were mainly enriched in amino sugar and nucleotide sugar metabolism, biosynthesis of unsaturated fatty acids, cardiac muscle contraction, chemokine signaling pathway, and Fc epsilon RI signaling pathway. The aforementioned signaling pathways and sepsis are also closely associated and are mainly related to the immune function of the cells (46–50).

To further explore the changes in immune cells in sepsis, we analyzed by scRNA-seq dataset, and the results showed that T/B/NK cells were lower in sepsis than in normal subjects, which is consistent with the findings of Wang et al. (51). These data provide ample evidence for the presence of significant immunosuppression in patients with sepsis. An in-depth analysis of the differences in immune cell abundance between the high- and low-risk groups for sepsis showed that CD4 memory activated T cells, Tregs, resting NK cells, M0 macrophages, and M2 macrophages were higher in the high-risk group than in the low-risk group. It was suggested that patients in the high-risk group showed a more significant reduction in cytotoxic function and a more active performance in the regulation of humoral immunity, fully reflecting the fact that sepsis is a complex mechanism of both transitional inflammation and immunosuppression. The main classes of drugs used for the evaluation of sepsis treatment are immunostimulatory drugs (immune targets CSF2RA/B, CSF3R) (52), immunostimulatory cytokines (immune target is IFNGR1/2) (53), and immunosuppressants (PDL1 and CTLA4) (54). We analyzed the immune targets of immune-related drugs to predict their potential use in sepsis and showed that nine target loci (CSF2RA/B, CSF3R, IFNGR1/2, IL7R, PDL1, CTLA4, and LAG3) differed in the high-/low-risk block. IL-7 is perhaps the most promising potential immunotherapy for sepsis (55). IL-7 acts extensively on cells of the adaptive immune system, promoting the proliferation and survival of primary and memory CD4+/CD8+ T cells (56), and can reverse the immune deficiency of sepsis (57).

Although our study has initially validated five prognostic signature genes as being elevated in sepsis, there are some limitations. First, new machine learning and artificial intelligence algorithms are increasingly being used in the diagnosis and prognosis of diseases; however, this study still uses classical methods to construct a prognostic model for sepsis (58, 59). Second, the expression and mechanism of action in predictive features in sepsis need to be further refined and validated experimentally. Third, the drug treatment corresponding to the immune target is yet to be confirmed by more clinical studies.

In summary, the prognostic signatures have good properties to predict the prognosis of patients with sepsis, and provides a reliable and precise basis for the possible mechanism and clinical treatment of the prognostic signature in sepsis by analyzing the prospect of immunotherapeutic targets for immunosuppressive-related drugs in sepsis. Our study validated prognostic signatures but failed to elucidate their expression in different immune cells. In future work, we will construct animal models of sepsis, validate the expression of prognostic signature genes in different immune cells, and further investigate the mechanisms of action of different genes in different immune cells to provide sufficient evidence for the treatment of sepsis.

## Data availability statement

The datasets presented in this study can be found in online repositories. The names of the repository/repositories and accession number(s) can be found in the article/**Supplementary Material**.

## Ethics statement

The studies involving human participants were reviewed and approved by Ethics Committee of Shenzhen hospital, Southern Medical University (Registration number: MCSC-20220909-0001). Written informed consent for participation was not required for this study in accordance with the national legislation and the institutional requirements.

## Author contributions

HH, TH, and YZ conceived and designed the study. FY, HWS, YW, and KC were responsible for data download, collection, and statistical analysis. SG, LZ, and JL completed the first draft; XT, HY, and HBS revised the manuscript; and the submitted version was reviewed by YZ. All authors contributed to the manuscript and approved the submitted version.

## Funding

Funding for this study was obtained from the Wu Jieping Medical Foundation (320.6750.2021-06-30), the Guangdong Basic and Applied Basic Research Foundation (2019A1515110120), the National Natural Science Foundation of China (82002974), and the Shenzhen Hospital of Southern Medical University, Research Promotion Funds for the Key Discipline Construction Program (ZDXKKYTS006).

## Acknowledgments

We thank all the healthcare professionals who participated in this study, as well as the investigators who provided free data and the GEO platform. Thanks to Dr. Hua Yan of the ICU for providing the diagnosis certificate of the sepsis patient.

## Conflict of interest

The authors declare that the research was conducted in the absence of any commercial or financial relationships that could be construed as a potential conflict of interest.

## Publisher’s note

All claims expressed in this article are solely those of the authors and do not necessarily represent those of their affiliated organizations, or those of the publisher, the editors and the reviewers. Any product that may be evaluated in this article, or claim that may be made by its manufacturer, is not guaranteed or endorsed by the publisher.

## Abbreviations

scRNA-seq: single-cell RNA sequencing; DEG: differentially expressed gene; GO: Gene Ontology; KEGG: Kyoto Encyclopedia of Genes and Genomes; GEO: Gene Expression Omnibus; BP: biological process; MF: molecular function; CC: cellular component; CCL5: C-C motif chemokine ligand 5; PCA: principal component analysis; GSEA: gene set enrichment analysis; non-ICU: non-intensive care unit; OS: overall survival; UMAP: Uniform Manifold Approximation and Projection; Tregs: T cells regulatory; SIRS: systemic inflammatory response syndrome; HbA2: hemoglobin subunit alpha 2; COVID-19; coronavirus disease 2019; LPS: lipopolysaccharide.
