# Supplementary material for: Identification of a novel sepsis prognosis model and analysis of possible drug application prospects: Based on scRNA-seq and RNA-seq data
Source: Front Immunol. 2022 Oct 28;13:888891. doi: 10.3389/fimmu.2022.888891 (PMC9650379; doi:10.3389/fimmu.2022.888891)
Supplement: Supplementary file 2 [file Table_1.docx]

**Supplementary Materials** 1: primer sequence.

| Genes | Primer Sequence（5' to 3'） |
| --- | --- |
| CCL5-F | GAAAGAACCGCCAAGTGTGT |
| CCL5-R | AGCTAGGACAAGAGCAAGCA |
| HBD-F | TGCTGTCAATGCCCTGTGGG |
| HBD-R | GGTTGTCCAGGTGAGCCAGG |
| IRF2BP2-F | ACCGCGTCGAGTTCGTCATC |
| IRF2BP2-R | GGATGTCCTTGGCGGAGAGC |
| LTB-F | GGCGGTGCCTATCACTGTCC |
| LTB-R | ACGCCTGTTCCTTCGTCGTC |
| WFDC1-F | ATGGCTTGGTGGCAATGGCT |
| WFDC1-R | CGGTTGGGGATACCTTCGGC |
| GAPDH-F | AGATCCCTCCAAAATCAAGTGG |
| GAPDH-R | GGCAGAGATGATGACCCTTTT |
